# Intermittent Fasting Alleviates the Increase of Lipoprotein Lipase Expression in Brain of a Mouse Model of Alzheimer's Disease: Possibly Mediated by β-hydroxybutyrate

**DOI:** 10.3389/fncel.2018.00001

**Published:** 2018-01-17

**Authors:** Jingzhu Zhang, Xinhui Li, Yahao Ren, Yue Zhao, Aiping Xing, Congmin Jiang, Yanqiu Chen, Li An

**Affiliations:** Department of Nutrition and Food Hygiene, School of Public Health, China Medical University, Shenyang, China

**Keywords:** Alzheimer's disease, intermittent fasting, β-hydroxybutyrate, lipoprotein lipase, microRNA-29a, histone deacetylases

## Abstract

Intermittent fasting has been demonstrated to protect against Alzheimer's disease (AD), however, the mechanism is unclear. Histone acetylation and lipoprotein lipase (LPL) are involved in AD progression. Importantly, LPL has been documented to be regulated by histone deacetylases (HDACs) inhibitors (increase histone acetylation level) in adipocyte and mesenchymal stem cells, or by fasting in adipose and muscle tissues. In brain, however, whether histone acetylation or fasting regulates LPL expression is unknown. This study was designed to demonstrate intermittent fasting may protect against AD through increasing β-hydroxybutyrate, a HDACs inhibitor, to regulate LPL. We also investigated microRNA-29a expression associating with regulation of LPL and histone acetylation. The results showed LPL mRNA expression was increased and microRNA-29a expression was decreased in the cerebral cortex of AD model mice (APP/PS1), which were alleviated by intermittent fasting. No significant differences were found in the total expression of LPL protein (brain-derived and located in capillary endothelial cells from peripheral tissues) in the cerebral cortex of APP/PS1 mice. Further study indicated that LPL located in capillary endothelial cells was decreased in the cerebral cortex of APP/PS1 mice, which was alleviated by intermittent fasting. LPL and microRNA-29a expression were separately increased and down-regulated in 2 μM Aβ_25−35_-exposed SH-SY5Y cells, but respectively decreased and up-regulated in 10 μM Aβ_25−35_-exposed cells, which were all reversed by β-hydroxybutyrate. The increase of HDAC2/3 expression and the decrease of acetylated H3K9 and H4K12 levels were alleviated in APP/PS1 mice by intermittent fasting treatment, as well in 2 or 10 μM Aβ_25−35_-exposed cells by β-hydroxybutyrate treatment. These findings above suggested the results from APP/PS1 mice were consistent with those from cells treated with 2 μM Aβ_25−35_. Interestingly, LPL expression was reduced (0.2-folds) and microRNA-29a expression was up-regulated (1.7-folds) in HDAC2-silenced cells, but respectively increased (1.3-folds) and down-regulated (0.8-folds) in HDAC3-silenced cells. Furthermore, LPL expression was decreased in cells treated with microRNA-29a mimic and increased with inhibitor treatment. In conclusion, intermittent fasting inhibits the increase of brain-derived LPL expression in APP/PS1 mice partly through β-hydroxybutyrate-mediated down-regulation of microRNA-29a expression. HDAC2/3 may be implicated in the effect of β-hydroxybutyrate on microRNA-29a expression.

## Introduction

Alzheimer disease (AD) is a common neurodegenerative disease, presenting a memory loss and other cognitive abilities serious enough to interfere with daily life. The disorder is morphologically characterized by extracellular deposition of amyloid-β (Aβ) combined with formation of neuritis spot and neurofibrillary tangles (NFT) as well as death and loss of cholinergic neurons (Whitehouse et al., 1982). The “Aβ cascade hypothesis” considers Aβ species to be the molecular triggers of a cascade of events (amyloid cascade) leading to synaptic dysfunction and neuronal loss (Hardy and Selkoe, 2002). Accordingly, targets reducing the brain Aβ burden may be alternative strategies for AD therapy and prevention.

Ketone bodies have demonstrated efficacy in animal models of neurodegenerative disorders and in human clinical trials, including AD trials (Henderson, 2008). Acetoacetate (AcAc) and beta-hydroxybutyrate (βOHB) account for the majority of ketone bodies (Robinson and Williamson, 1980). AcAc and βOHB is a redox pair, which can be converted to each other based on the redox state of the nicotinamide adenine dinucleotide (NAD)/NADH couple (Laffel, 1999). Ketone bodies are normally produced by the liver from fatty acids when glucose supplies are limited, such as an acute fasting (Cahill et al., 1966) or intermittent fasting (Wang et al., 2017). Intermittent fasting is an umbrella term for various diets that cycle between a period of fasting and non-fasting. Notably, researchers have found that intermittent fasting plays a role in forestalling the onset of certain neurodegenerative disorders, including AD (Stewart et al., 1989; Halagappa et al., 2007). Our previous study has also shown that intermittent fasting can improve cognitive function and protect against brain Aβ deposition in the APP/PS1 transgenic mouse model of AD (Zhang et al., 2017b). From the above, ketone bodies may be implicated in the anti-AD effect of intermittent fasting. What's important, about 70% of ketone bodies are βOHB (Robinson and Williamson, 1980), which has been reported to provide protection against AD (Xie et al., 2015; Cunnane et al., 2016). Therefore, βOHB may play a major role in the protection of intermittent fasting against AD. Moreover, as an endogenous pan-histone deacetylases (HDACs) inhibitor, βOHB can suppress HDAC2 and HDAC3 (Shimazu et al., 2013). Accumulating evidence has indicated that the levels of histone acetylation are significantly reduced and the expression of HDAC2/3 is remarkably increased in the brain of AD (Xu et al., 2011; Zhang et al., 2017a). However, it is still unknown whether βOHB participates in the anti-AD effect of intermittent fasting through affecting the levels of histone acetylation to regulate AD-related gene expression.

Lipoprotein lipase (LPL) is a member of the lipase gene family, which most widely distributes in adipose, heart, and skeletal muscle tissue, as well as in brain (Merkel et al., 2002). Notably, LPL protein located in the brain capillary endothelial cells is thought to be derived from peripheral tissue (Bessesen et al., 1993). The genetic polymorphism of LPL has been found to be closely related to the onset of AD (Gong et al., 2013). LPL has also been documented to accumulate in senile plaques of AD brains (Rebeck et al., 1995), and as a molecular chaperone to bind to Aβ (Nishitsuji et al., 2011). As a key enzyme in lipoprotein metabolism, LPL has been found to be increased in adipose and decreased in muscle tissue by fasting, exhibiting tissue specificity (Goldberg et al., 2009). However, it is not clear the effect of intermittent fasting on the regulation of LPL expression in brain. Furthermore, the expression of LPL was increased in adipose cells by treatment with a HDACs inhibitor diallyl disulfide (Lee et al., 2007), but decreased in mesenchymal stem cells treated by sodium butyrate, another HDACs inhibitor (Chen et al., 2007). In the context of previous observations, this study investigated whether intermittent fasting up-regulates or down-regulates the expression of LPL by βOHB-mediated inhibition of HDACs in AD brain.

In oxLDL-stimulated dendritic cells, the expression of LPL was repressed by microRNA-29a (miR-29a) (Chen et al., 2011). MicroRNAs, a novel class of short (~22 nucleotides) non-coding RNAs, are identified as important post-transcriptional inhibitors of gene expression by base pairing with the 3′untranslated regions (UTRs) of mRNAs and promoting mRNA stability (Chen et al., 2011; Ribeiro et al., 2014). The expression of microRNAs can also be regulated by histone acetylation. However, it is unknown whether miR-29a is implicated in HDACs-mediated regulation of LPL expression in the effect of βOHB against AD.

In the present study, we found that intermittent fasting inhibited the increase of brain-derived LPL expression in AD, which was partly mediated by βOHB. Furthermore, miR-29a was found to mediate the effect of βOHB on LPL expression, which HDAC2/3 may be implicated in the regulation of miR-29a expression by βOHB. Importantly, our findings indicated that in SH-SY5Y cells, different concentrations of Aβ (2 or 10 μM) have opposite effects on the regulation of LPL expression, as well miR-29a, which were reversed by βOHB. The expression of LPL was reduced and miR-29a was up-regulated in HDAC2-silenced SH-SY5Y cells; however, silencing HDAC3 has opposite effects on the regulation of LPL and miR-29a expression.

## Materials and methods

### Reagents

βOHB was purchased from Shanghai Yingxin Laboratory Equipment Co., Ltd. (Shanghai, China); rabbit anti-β-actin polyclonal antibody (sc-130656), rabbit anti-HDAC2 polyclonal antibody (sc-7899), rabbit anti-HDAC3 polyclonal antibody (sc-11417) and rabbit anti-LPL polyclonal antibody (sc-32885)were purchased from Santa Cruz Biotechnology (Santa Cruz, California); rabbit anti-acetylation of histone 3 lysine 9 (Ace-H3K9) polyclonal antibody (YK0006), rabbit anti-Ace-H4K12 polyclonal antibody (YK0013) and rabbit anti-Histone H3.1 polyclonal antibody (YK0009) were purchased from ImmunoWay Inc. (USA). Rat anti-mouse CD31 monoclonal antibody (550274) was obtained from BD Biosciences (USA). Goat anti-rabbit immunoglobulin G (lgG) secondary antibody FITC-donkey anti-rabbit IgG secondary antibody and CY3-donkey anti-rat lgG secondary antibody were from Shanghai Sangon Biotech Co., Ltd. (Shanghai, China). Cell culture medium was purchased from American Hyclone Inc. (USA). Aβ_25−35_ was obtained from American Peptide Inc. (USA), which is a toxic fragment of the full-length Aβ peptide. Before use, Aβ_25−35_ was solubilized in sterile water and aggregated at 37°C for 7 d. Immunohistochemistry kits were obtained from Beijing Zhongshan Biotechnology (Beijing, China).

### *In vivo* study

#### Animals and treatment

The animals breeding and treatment were performed as previously described (Zhang et al., 2017b). Briefly, APP/PS1 double-transgenic mice [B6C3-Tg (APPswe, PS1dE) 85Dbo/J] and wild-type littermates were obtained from Jackson Laboratory (USA) and housed in the temperature-controlled 12 h-light/l2 h-dark environment. Protocols were approved by the Animal Care and Use Committee of China Medical University. In this study, alternate-day fasting (ADF) was used as a means of intermittent fasting, namely mice were fed *ad libitum* every other day (24 h) and fasted the following day (24 h). Our preliminary experiment found the level of βOHB in blood increased in C57BL/6 mice after 12 h or longer fasting, however, no significant change in the blood βOHB level at 8 h in mice after fasting treatment. Accordingly, ADF can cause a regularly fluctuant change in the level of βOHB in mice. At 5 months of age, wild-type mice were divided into 2 groups: WT and WT+ADF, and APP/PS1 mice were divided into 2 groups: AD and AD+ADF. Mice in WT and AD groups were fed *ad libitum* and in WT+ADF and AD+ADF groups were treated by ADF. Each group had 5 males and 5 females, with roughly balanced body weights across the groups. After 5 months, the mice were fasted for 8 h and then sacrificed under ether anesthesia. Their brains were collected, weighed and divided into halves, which the left hemi brain was used for immunofluorescence test and the right was stored at −80°C and then used for quantitative reverse transcriptase (qRT)-polymerase chain reaction (PCR) and Western blot analyses.

#### Immunofluorescence

Immunofluorescence was performed for LPL and CD31 staining with the same method as previously described until incubated with primary antibody (Zhang et al., 2017a). Then sections were incubated with primary rabbit anti-LPL polyclonal antibody (1:200) and rat anti-mouse CD31 monoclonal antibody (1:30) at 4°C overnight, subsequently, incubated in dark with FITC-coupled donkey anti-rabbit secondary antibody and CY3-donkey anti-rat IgG secondary antibody for 30 min. DAPI was used as a nuclear stain, then washed and finally mounted in glycerol, containing 1% n-propyl gallate. Sections were observed under a Fluorescence Microscope (Nikon 80i, Japan) and photographed. Images were compounded using FV10-ASW 2.1 Viewed software. Five visual fields were selected in each slice to obtain the fluorescence intensity by using Image J software. The result was expressed in average fluorescence intensity to assess the protein expression level.

#### qRT-PCR assay of cortex samples

Total mRNA extraction and reverse transcription were performed with the same method as our previously described (Zhang et al., 2017a). MicroRNAs were isolated from thawed cerebral cortex with San Prep Column microRNA Mini-Preps Kit (Shanghai Sangon Biotech Co., Ltd., China). MicroRNAs reverse transcription was performed by microRNAs First Strand cDNA Synthesis (Shanghai Sangon Biotech Co., Ltd., China). After complementary DNAs synthesis, all Real-Time PCR reactions were performed as we have described previously (Zhang et al., 2017a). The following primer sequences were used: mus LPL, forward: 5′-CCAAGAGAAGCAGCAAGATGTA-3′, reverse: 5′-ATCCTCAGTCCCAGAAAAGTGA-3′ (123 bp product); mus HDAC2, forward: 5′-GCCAAGTCAGAACAACTCAGC-3′, reverse: 5′-GTCCTCAAACAGGGAAGGTT-3′ (104 bp product); mus HDAC3, forward: 5′-ATCCGCCAGACAATCTTTGA-3′, reverse: 5′-CTCGGGACCTCTCTCTTCAG-3′ (132 bp product); β-actin forward: 5′-CATCCGTAAAGACCTCTATGCCAAC-3′, reverse: 5′-ATGGAGCCACCGATCCACA-3′ (171 bp product); mmu-miR-29a-3p, forward: 5′-GTAGCACCATCTGAAATCGGTTA-3′, reverse: 5′-CGCTTCACGAATTTGCGTGTCAT-3′; U6, forward: 5′-GCTTCGGCAGCACATATACTAAAAT-3′, reverse: 5′-CGCTTCACGAATTTGCGTGTCAT-3′. Results were expressed relative to β-actin mRNA or U6 used as an internal control (*n* = 10). Comparative C_T_ method (also known as the 2^−ΔΔ*CT*^ method) was used to analyze data.

#### Western blot analysis of cortex samples

Western blot was performed as previously reported (Zhang et al., 2017a). Briefly, RIPA buffer containing 0.1% protease inhibitor (Amerso, USA) was used to homogenize thawed cerebral cortex samples. Protein concentrations in the supernatants were measured by Bradford method with Coomassie Brilliant Blue (CBB G250) and bovine serum albumin as a standard. Equal amounts of soluble protein (40 μg) were used for Western blot test, using rabbit anti-LPL (1:1,000), anti-Ace-H3K9 (1:200), anti-Ace-H4K12 (1:200), anti-Histone H3.1 (1:200), anti-HDAC2 (1:1,000), anti-HDAC3 (1:1,000), or anti-β-actin (1:1,000) antibody. β-actin or Histone H3.1 was used as a reference standard. The results for western blot were expressed as folds of WT.

### *In vitro* study

#### Cell culture

Human SH-SY5Y neuroblastoma cells (The Chinese academy of sciences cell bank, KCB2006107YJ, Kunming, China) were cultured in DMEM/F12 (1:1) media with 10% fetal bovine serum, 100 U/ml penicillin, and 100 μ g/ml streptomycin in an incubator at 37°C with 5% CO_2_ as described previously (Zhang et al., 2017a). The cells used in each experimental group were at passage 5.

#### qRT-PCR and western blot analyses of cells

Cells were incubated in six-well culture microplates in antibiotic-free medium with (βOHB and βOHB+Aβ groups) or without (control and Aβ groups) βOHB (final concentration 2 mM) for 3 h; the βOHB concentration was selected based on the results of previous MTS [3-(4,5-Dimethylthiazol-2-yl)-5-(3-carboxymethoxyphenyl)-2-(4-sulfophenyl)-2H-tetrazolium] assay and the obtainable level of βOHB *in vivo* (Robinson and Williamson, 1980). Three hours later, the cultures in Aβ and βOHB+Aβ groups were quickly mixed with Aβ_25−35_ (final concentration 2 or 10 μM) for an additional 12 h culture. Subsequently, the mRNA, microRNA and protein expression in the cells were measured by qRT-PCR and Western blot, as described above. The following primer sequences were used: homo LPL, forward: 5′-CCGCCGACCAAAGAAGAGAT-3′, reverse: 5′-TAGCCACGGACTCTGCTACT-3′ (117 bp product); homo HDAC2 forward: 5′-AGGTTGAAGCCATTCTCCTG-3′, reverse: 5′-ATCCCAGCACTTTGGAAGG-3′ (179 bp product); homo HDAC3 forward: 5′-GAGGGATGAACGGGTAGACA-3′, reverse: 5′-CAGGTGTTAGGGAGCCAGAG-3′ (137 bp product); β-actin, forward: 5′-CATCCGTAAAGACCTCTATGCCAAC-3′, reverse: 5′-ATGGAGCCACCGATCCACA-3′ (171 bp product); hsa-miR-29a-3p, forward: 5′-CTAGCACCATCTGAAATCGGTTA-3′, reverse: 5′-CGCTTCACGAATTTGCGTGTCAT-3′; U6, forward: 5′-GCTTCGGCAGCACATATACTAAAAT-3′, reverse: 5′-CGCTTCACGAATTTGCGTGTCAT-3′. Levels of proteins were analyzed with the corresponding primary antibodies: anti-LPL (1:1,000), anti-Ace-H3K9 (1:200), anti-Ace-H4K12 (1:200), anti-Histone H3.1 (1:200), anti-HDAC2 (1:1,000), anti-HDAC3 (1:1,000), or anti-β-actin (1:1,000) antibody. The results for western blot were expressed as folds of control. This experiment was repeated three times and conducted in duplicate.

#### Transfection of microRNA mimic and inhibitor

micrOFF® miRNA mimic and inhibitor for human miR-29a were designed and synthesized by Guangzhou RiboBio Co., Ltd. (Guangzhou, China). miR-29a mimic sequence: 5′-UAGCACCAUCUGAAAUCGGUUA-3′, anti-sequence: 5′-AUCGUGGUAGACUUUAGCCAAU-3′; and miR-29a inhibitor sequence: 5′-mUmAmAmCmCmGmAmUmUmUmCmAmGmAmUmGmGmUmGmCmUmA-3′ (mN, 2′-O-methyl ribose). Cells were transfected in six-well culture microplates with 200 nM miRNA mimic or inhibitor according to the manufacturer's protocol (ribo FECT™ CP Transfection Kit; Guangzhou RiboBio CO, LTD) for 24 hat 37°C prior to measurements in antibiotic-free medium. micrOFF® miRNA mimic control and micrOFF® miRNA inhibitor control (Guangzhou RiboBio Co., Ltd.) were used as controls, respectively. Subsequently, the cells were collected and total mRNA, microRNA and protein were extracted. In addition, the mRNA and protein expression levels of LPL were investigated by the above methods. The results for western blot were expressed as folds of mimic control or inhibitor control. The experiment was repeated three times and performed in duplicate.

#### Small interfering RNA (siRNA)

The endogenous HDAC2 and HDAC3 mRNA levels in cells were interfered by HDAC2 and HDAC3 siRNA duplex (Guangzhou RiboBio CO, LTD), respectively. The following siRNA oligos were used: HDAC1: 5′-CCGGTCATGTCCAAAGTAA-3′; HDAC2: 5′-TCCGTAATGTTGCTCGATG-3′; HDAC3: 5′-GCATTGATGACCAGAGTTA-3′. siRNA was performed as we have described in detail previously (Zhang et al., 2017a). qRT-PCR and Western blot analyses were used to evaluate the interference efficiency. LPL mRNA and protein expression and miR-29a expression were measured by the above methods. The results for western blot were expressed as folds of scrambled siRNA. The experiment was repeated three times and conducted in duplicate.

### Statistical analyses

Statistical analysis of the data was performed by one-way analyses of variance (ANOVAs) including appropriate variables followed by Fisher's least significant difference (LSD) multiple comparison *post-hoc* tests. Data are presented graphically as means ± standard deviations (SDs). Results were considered significant when *p* < 0.05.

## Results

### ADF alleviated the increase of LPL mRNA expression in the cerebral cortex of AD model mice

**Figure 1:**
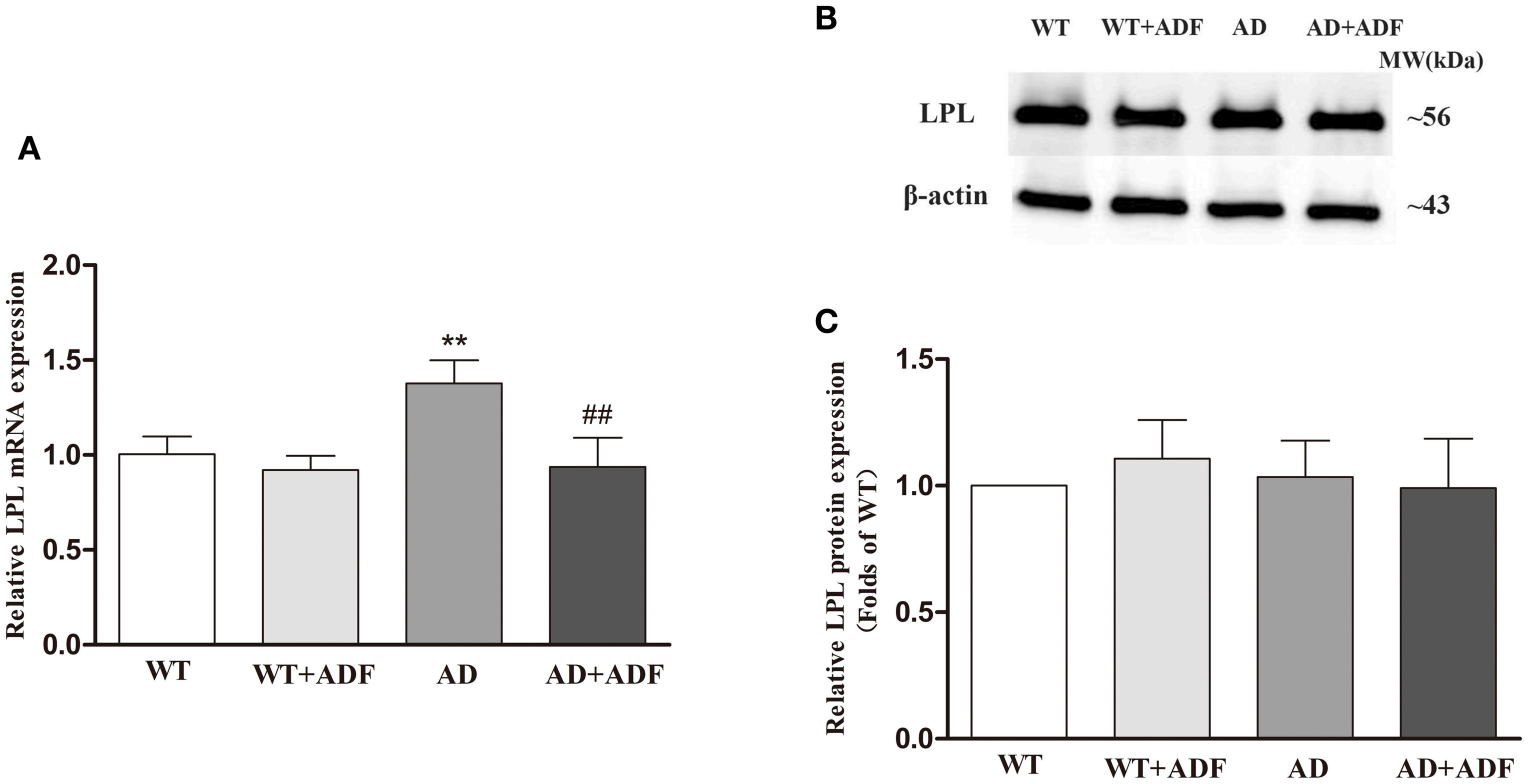
ADF alleviated the increase of LPL mRNA expression in the cerebral cortex of AD model mice. The relative expression of LPL mRNA (**A**; β-actin as a reference standard) and protein **(B,C)** were analyzed by qRT-PCR and Western blot, respectively (*n* = 10; mean ± SD; One-way ANOVA followed by LSD multiple comparison tests; ^**^p < 0.01 vs. WT group, ^##^*p* < 0.01 vs. AD group).

To investigate the alteration of LPL in the effect of ADF against AD, the expression of LPL mRNA (Figure 1A) and protein (Figures 1B,C) in the cerebral cortex was measured by qRT-PCR and Western blot, respectively. In AD model mice, the mRNA expression of LPL was significantly increased (*p* < 0.01) relative to the expression observed in WT mice. Meanwhile, compared with AD model mice, the expression of LPL mRNA was significantly decreased (*p* < 0.01) in the ADF-treated AD model mice. However, no significant differences were found in protein expression of LPL among groups.

### ADF alleviated the decrease of LPL protein expression in the capillary endothelial cells of cerebral cortex in AD model mice

**Figure 2:**
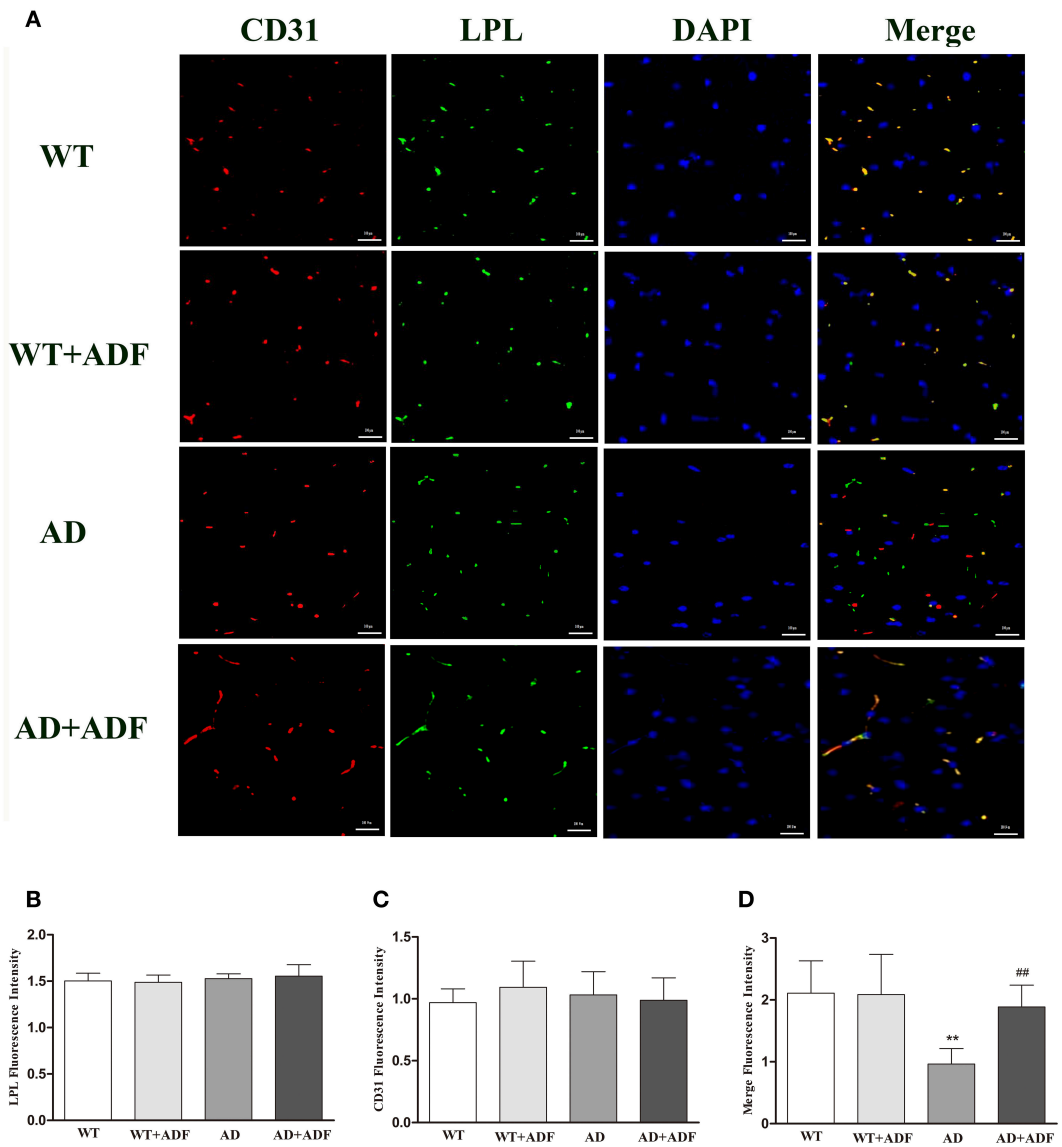
ADF alleviated the decrease of LPL protein expression in the capillary endothelial cells of cerebral cortex in AD model mice. **(A)** Immunofluorescence analysis was performed using anti-LPL (green) and anti-CD31 (red) antibodies, and cell nuclei were counterstained with DAPI (blue) (bars = 100 μm). **(B)** The LPL fluorescence intensity. **(C)** The CD31 fluorescence intensity. **(D)** The merge of LPL and CD31 fluorescence intensity (*n* = 10; mean ± SD; One-way ANOVA followed by LSD multiple comparison tests; ^**^p < 0.01 vs. WT group, ^##^*p* < 0.05 vs. AD group; magnify 100×).

Immunofluorescence was performed for LPL (green) and CD31 (red) staining to examine the expression of LPL located in capillary endothelial cells of the cerebral cortex in mice (Figure 2). Immunoflorescent labeling demonstrated that there were no obvious differences (*p* > 0.05) in the expression of LPL and CD31 among groups. Compare with WT mice, a low level (*p* < 0.01) of colocalization (yellow) in fluorescent staining of LPL and CD31 occurred in AD model mice, while in AD model mice treated with ADF, the colocalization of fluorescent signal was strongly increased (*p* < 0.01) relative to AD model mice.

### ADF alleviated the decrease of Ace-H3K9 and Ace-H4K12 levels in the cerebral cortex of AD model mice

**Figure 3:**
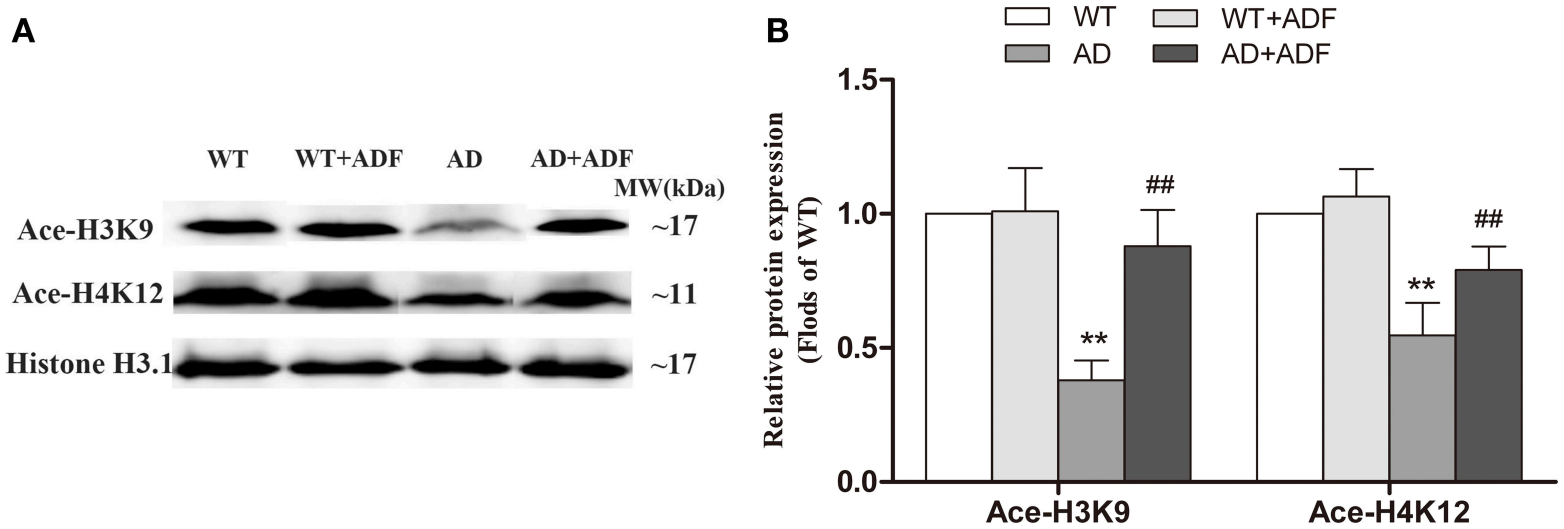
ADF alleviated the decrease of Ace-H3K9 and Ace-H4K12 levels in the cerebral cortex of AD model mice. Western blot was used to analyze the relative protein expression of Ace-H3K9 and Ace-H4K12 **(A,B)** (*n* = 10; mean ± SD; One-way ANOVA followed by LSD multiple comparison tests; ^**^p < 0.01 vs. WT group, ^##^*p* < 0.01 vs. AD group).

To explore the mechanisms on the regulation of LPL expression by ADF in AD model mice, we investigated the levels of Ace-H3K9 and Ace-H4K12 in the cerebral cortex among groups (Figure 3). In AD model mice, the levels of Ace-H3K9 and Ace-H4K12 were significantly decreased (*p* < 0.01) relative to the levels observed in WT mice. Additionally, compared with AD model mice, the levels of Ace-H3K9 and Ace-H4K12 were obviously increased (*p* < 0.01) in AD model mice treated with ADF.

### ADF alleviated the increase of HDAC2/3 mRNA and protein expression as well as the decrease of miR-29a expression in the cerebral cortex of AD model mice

**Figure 4:**
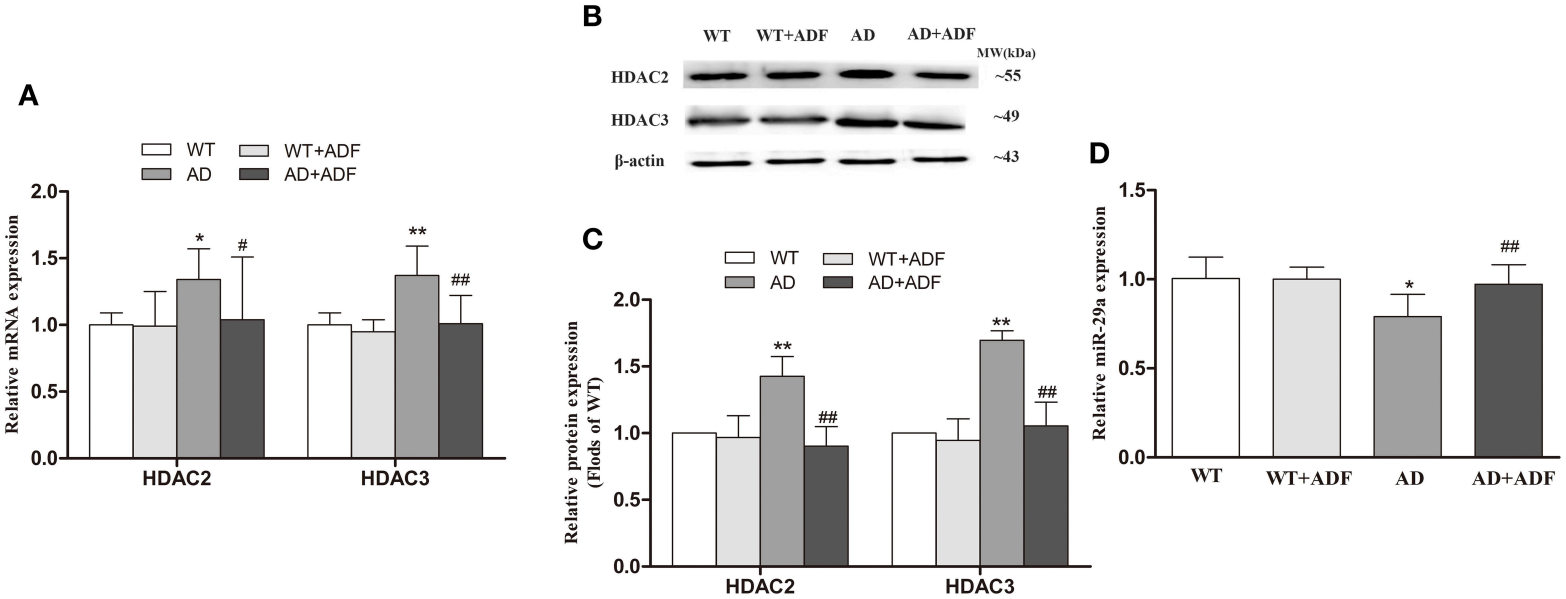
ADF alleviated the increase of HDAC2/3 expression and the decrease of miR-29a expression in the cerebral cortex of AD model mice. The relative expression of HDAC2/3 mRNA (**A**; β-actin as a reference standard) and protein **(B,C)** and miR-29a (**D**; U6 as a reference standard) were analyzed by qRT-PCR and Western blot, respectively (*n* = 10; mean ± SD; One-way ANOVA followed by LSD multiple comparison tests; ^*^p < 0.05, ^**^p < 0.01 vs. WT group, ^#^*p* < 0.05, ^##^*p* < 0.01 vs. AD group).

To further investigate the regulation of ADF on LPL expression in AD model mice, we examined the expression of HDAC2/3 (Figures 4A–C) and miR-29a (Figure 4D) in the cerebral cortex among groups. Compared with WT mice, the expression of HDAC2/3 mRNA and protein was significantly increased (*p* < 0.05; *p* < 0.01) and the expression of miR-29a was significantly decreased (*p* < 0.05) in AD model mice. After ADF intervention, an evident decrease in HDAC2/3 expression (*p* < 0.05; *p* < 0.01) and a marked increase in miR-29a expression (*p* < 0.01) were found in AD model mice.

### The effect of βOHB on LPL expression in Aβ-exposed SH-SY5Y cells

**Figure 5:**
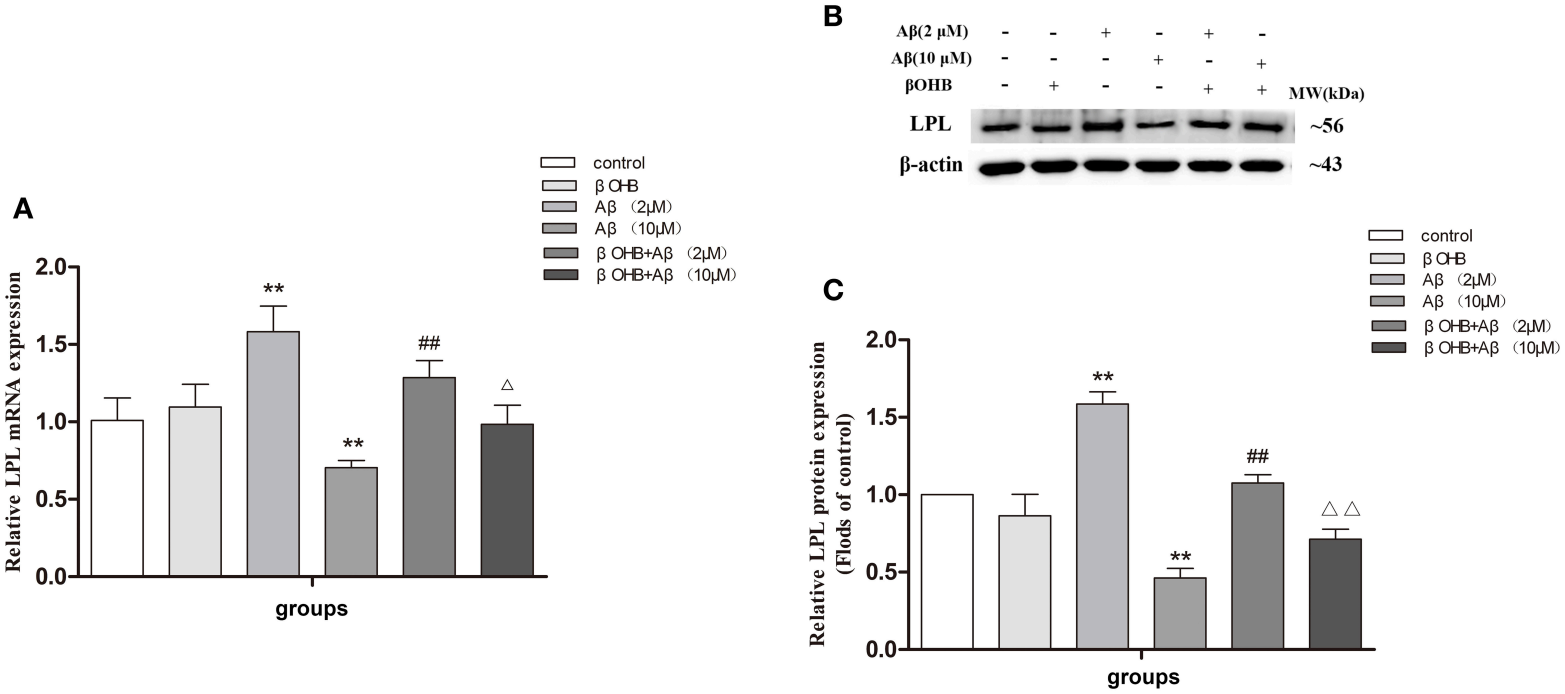
The effect of βOHB on LPL expression in Aβ-exposed SH-SY5Y cells. The relative expression of LPL mRNA (**A**; β-actin as a reference standard) and protein **(B,C)** were analyzed by qRT-PCR and Western blot, respectively (*n* = 6; mean ± SD; One-way ANOVA followed by LSD multiple comparison tests; ^**^p < 0.01 vs. control group, ^##^*p* < 0.01 vs. Aβ (2 μM) group, ^Δ^*p* < 0.05, ^ΔΔ^*p* < 0.01 vs. Aβ (10 μM) group).

It is known that ADF results in an increase of endogenous βOHB, and our preliminary experiment also showed that the level of βOHB increased strikingly after fasting for 12 h (data unlisted). To investigate whether βOHB mediates the regulation of LPL expression in the anti-AD effect of ADF, we detected the expression of LPL in Aβ-treated cells with or without βOHB pretreatment (Figure 5). Compared with control, the expression of LPL mRNA and protein was significantly increased (*p* < 0.01) in cells exposed to 2 μM Aβ, but obviously decreased (*p* < 0.01) in cells exposed to10 μM Aβ. The expression of LPL mRNA and protein was remarkably decreased in βOHB-pretreated cells exposed to 2 μM Aβ, and significantly increased in βOHB-pretreated cells exposed to 10 μM Aβ, relative to the expression observed in cells treated with Aβ only (*p* < 0.05; *p* < 0.01).

### βOHB prevented from Aβ-induced decrease in Ace-H3K9 and Ace-H4K12 protein levels in SH-SY5Y cells

**Figure 6:**
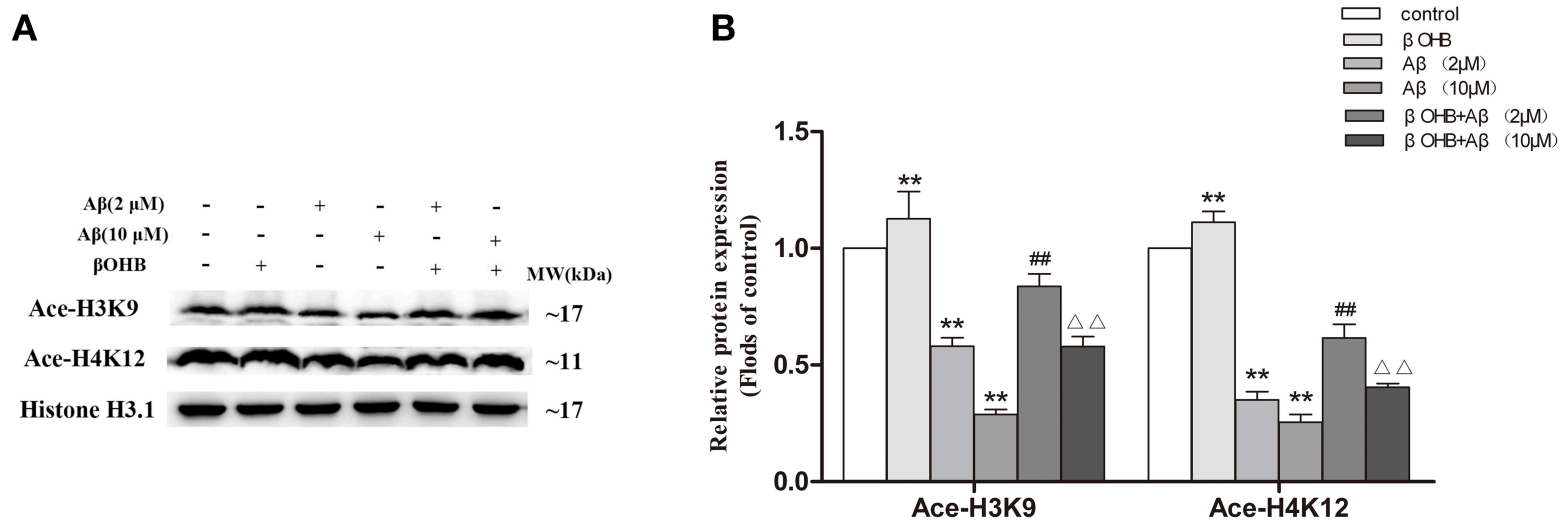
βOHB prevented from Aβ-induced decrease in Ace-H3K9 and Ace-H4K12 protein levels in SH-SY5Y cells. Western blot was used to analyze the relative protein expression of Ace-H3K9 and Ace-H4K12 **(A,B)** (*n* = 6; mean ± SD; One-way ANOVA followed by LSD multiple comparison tests; ^**^p < 0.01 vs. control group, ^##^*p* < 0.01 vs. Aβ (2 μM) group, ^ΔΔ^*p* < 0.01 vs. Aβ (10 μM) group).

As shown in Figure 6, compared with control, the levels of Ace-H3K9 and Ace-H4K12 were significantly decreased (*p* < 0.01) in cells with Aβ (2 or 10 μM) treatment, and strongly increased (*p* < 0.01) in βOHB-pretreated cells without Aβ treatment. Moreover, the marked increase (*p* < 0.01) of Ace-H3K9 and Ace-H4K12 protein levels were observed in Aβ-exposed (2 or 10 μM) cells in the presence of βOHB relative to cells exposed to Aβ (2 or 10 μM).

### βOHB alleviated the increase of HDAC2/3 mRNA and protein expression and the decrease of miR-29a mRNA expression in Aβ-exposed SH-SY5Y cells

**Figure 7:**
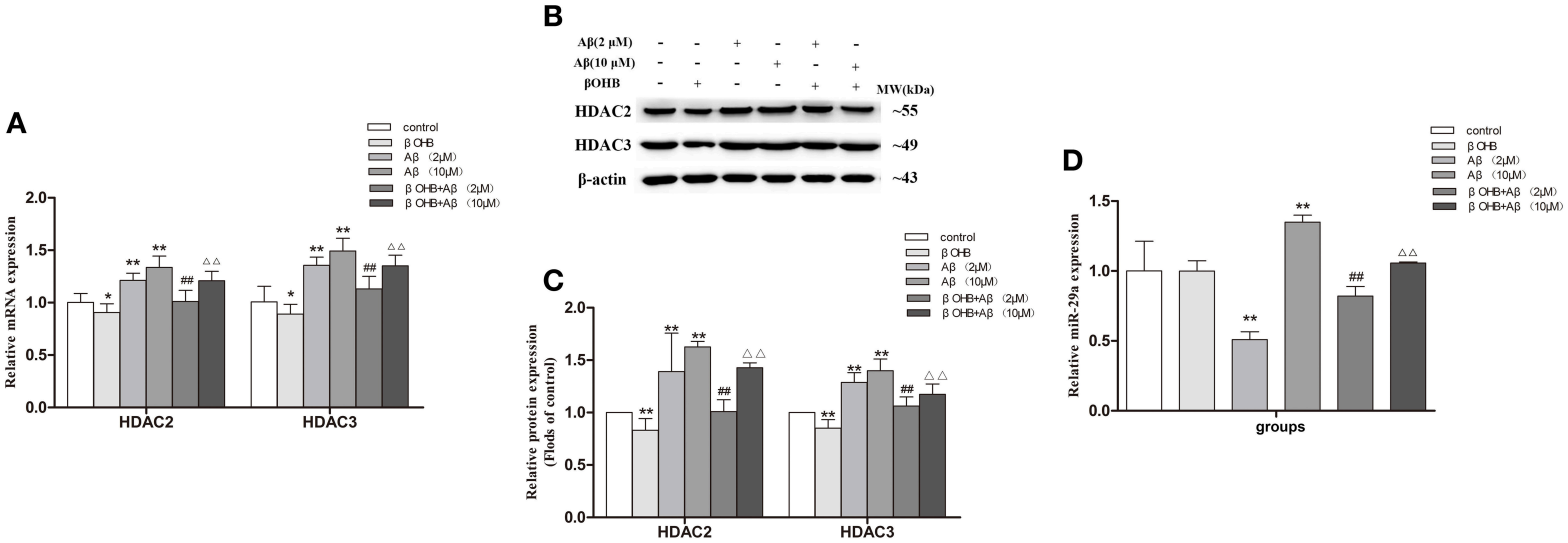
βOHB alleviated the increase of HDAC2/3 expression and the decrease of miR-29a expression in Aβ-exposed SH-SY5Y cells. The relative expression of HDAC2/3 mRNA (**A**; β-actin as a reference standard) and protein **(B,C)**, and miR-29a (**D**; U6 as a reference standard) were analyzed by qRT-PCR and Western blot, respectively (*n* = 6; mean ± SD; One-way ANOVA followed by LSD multiple comparison tests; ^*^p < 0.05, ^**^p < 0.01 vs. control group, ^##^*p* < 0.01 vs. Aβ (2 μM) group, ^Δ^*p* < 0.05, ^ΔΔ^*p* < 0.01 vs. Aβ (10 μM) group).

Cells exposed to 2 or 10 μM Aβ had marked increases in HDAC2/3 mRNA and protein expression (Figures 7A–C) and a significant decrease in miR-29a (Figure 7D) expression compared with those observed in control (*p* < 0.01). The decrease of HDAC2/3 mRNA and protein expression and the increase of miR-29a expression were observed in βOHB-pretreated cells compared with control (*p* < 0.05; *p* < 0.01). βOHB-pretreated cells exposed to 2 or 10 μM Aβ had an obvious decrease in HDAC2/3 expression and a significant increase (*p* < 0.01) in miR-29a expression compared with the levels observed in 2 or 10 μM Aβ-exposed cells.

### The effect of miR-29a on expression of LPL in SH-SY5Y cells

**Figure 8:**
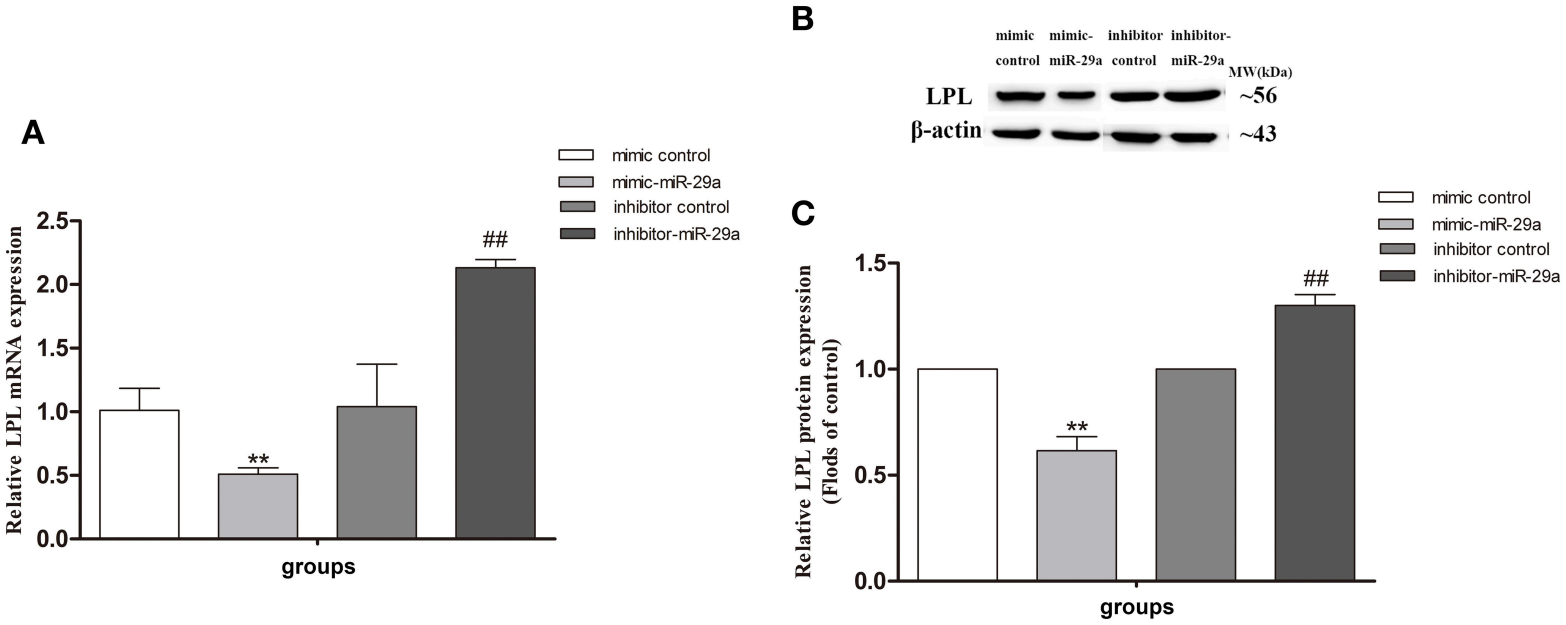
The effect of miR-29a on the expression of LPL in SH-SY5Y cells. The relative expression of LPL mRNA (**A**; β-actin as a reference standard) and protein **(B,C)** were analyzed by qRT-PCR and Western blot, respectively, in cells treated with miR-29a mimic and inhibitor (*n* = 6; mean ± SD; One-way ANOVA followed by LSD multiple comparison tests; ^**^p < 0.01 vs. mimic control group, ^##^*p* < 0.01 vs. inhibitor control group).

The miR-29a mimic and inhibitor were used to further investigate whether miR-29a regulates the LPL expression in SH-SY5Y cells (Figure 8). We found that the expression of LPL mRNA and protein was significantly decreased in cells treated with miR-29a mimic (*p* < 0.01) and was obviously increased in cells treated with miR-29a inhibitor (*p* < 0.01).

### Alteration of LPL and miR-29a expression in HDAC2- and HDAC3-silenced SH-SY5Y cells

**Figure 9:**
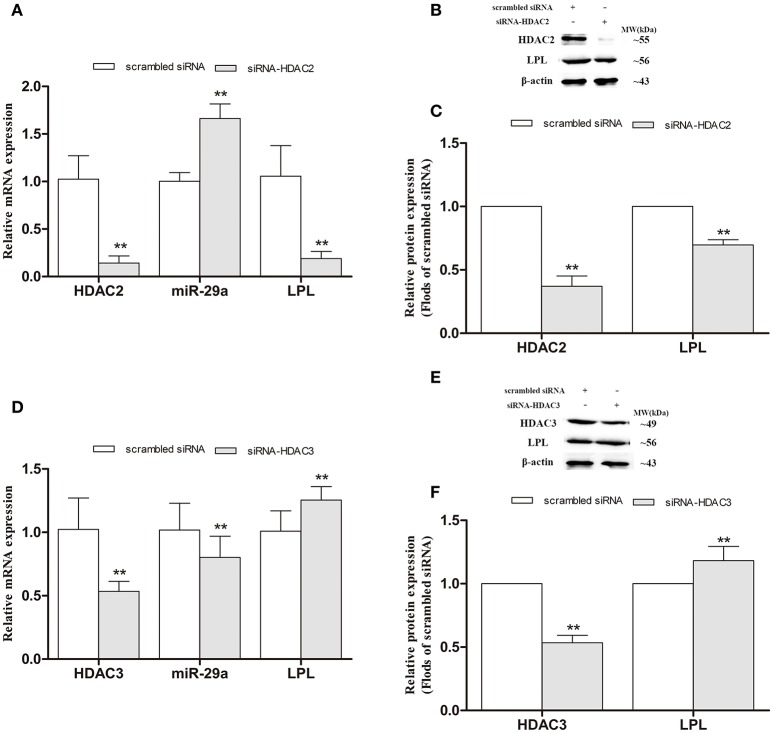
Alteration of LPL and miR-29a expression in HDAC2- and HDAC3-silenced SH-SY5Y cells. siRNA duplex was used to interfere with endogenous HDAC2/3 expression (nonspecific siRNA as controls). The relative expression levels of miR-29a (**A,D**; U6 as a reference standard), and LPL mRNA (**A,D**; β-actin as a reference standard) and protein **(B,C,E,F)** expression were analyzed by qRT-PCR and Western blot, respectively, in HDAC2- and HDAC3-silenced cells (*n* = 6; mean ± SD; One-way ANOVA followed by LSD multiple comparison tests; ^**^p < 0.01 vs. scrambled siRNA group).

To further determinate whether HDAC2/3 regulates LPL and miR-29a expression, the endogenous HDAC2 and HDAC3 mRNA expression levels in cells were interfered by HDAC2 and HDAC3 siRNA duplex (Figure 9). As illustrated in Figure 9A, compared with scrambled cells, the expression of miR-29a was significantly increased in HDAC2-silenced cells and obviously decreased in HDAC3-silenced cells (*p* < 0.01). In HDAC2-silenced cells, the expression of LPL was strongly decreased (*p* < 0.01), but obviously increased (*p* < 0.01) in HDAC3-silenced cells, compared with the cells exposed to the scrambled siRNA construct (scrambled cells).

## Discussion

Accumulating evidence has indicated that LPL is implicated in the progression of AD (Baum et al., 2000; Wang and Eckel, 2012). In human studies, the expression of LPL mRNA was increased in the brain of AD patients (Rebeck et al., 1995; Blain et al., 2006). The present study also showed a significant increase in LPL mRNA expression in the cerebral cortex of AD model mice; however, there were no obvious changes in protein expression level of LPL among groups by Western blot or immunofluorescence staining. LPL is synthesized and secreted into the surrounding interstitial spaces by cells (Davies et al., 2010). Subsequently, LPL is transported into the lumen of capillaries by the protein glycosylphosphatidylinositol-anchored high-density lipoprotein-binding protein 1 (GPIHBP1) which is expressed in endothelial cells and then bands with endothelial cell surface heparan sulfated proteoglycans (Bessesen et al., 1993; Davies et al., 2010). Since the distribution of GPIHBP1 in the brain capillaries is extremely sparse, LPL protein located in the brain capillaries endothelial cells is thought to be derived from peripheral tissue (Bessesen et al., 1993). In brief, LPL protein in brain tissue derives from not only brain itself but also peripheral tissue which is located in brain capillaries endothelial cells. In this study, the expression of LPL protein located in capillaries endothelial cells was reduced in the cerebral cortex of AD model mice. Considering no obvious difference in the total expression of LPL protein, the expression of brain-derived LPL protein was possibly increased in AD model mice, which is in line with the mRNA expression of LPL. Further study is still needed to explore the underlying mechanisms of down-regulation in LPL protein expression in capillaries endothelial cells of cerebral cortex in AD model mice. Furthermore, ADF was found to play a role in protecting against the increase of LPL mRNA and brain-derived LPL protein expression in the cerebral cortex of AD model mice, indicating that LPL may participate in the effect of ADF on AD.

ADF has been reported to increase the serum level of βOHB (Anson et al., 2003; Wang et al., 2017). Our preliminary experiment found the level of βOHB in blood increased after 12h or longer fasting in C57BL/6 mice. Tadahiro et al found βOHB concentration increased to about 10-fold in the serum of C57BL/6 mice after a 24-h fasting (Shimazu et al., 2013). Therefore, ADF may cause a regularly fluctuant change in the level of βOHB in mice. Furthermore, Anson et al investigated serum levels of βOHB after 14 h fasting in C57BL/6 mice fed *ad libitum* and mice treated with ADF for 20 weeks, and the results showed the serum level of βOHB increased to 2-fold in mice treated with ADF compared with mice fed *ad libitum* (Anson et al., 2003). These indicated that intermittent fasting may be different in metabolism with an acute fasting. Also, βOHB has been reported to take effect in delaying AD progression in several studies (Findlay et al., 2015; Xie et al., 2015). Accordingly, βOHB may play a crucial role in protective function of ADF against AD. In our *in vitro* study, we found that βOHB inhibited the increase of LPL expression in cells exposed to low concentrations of Aβ (2 μM), in agreement with the results from *in vivo* study. These suggested that βOHB may mediate, at least partly, the effect of ADF on the reduction of brain-derived LPL expression in AD. Interestingly, the expression of LPL was found to be decreased in cells exposure to high concentrations of Aβ (10 μM) which was also reversed by βOHB. Gong et al. (2013) reported that the expression of LPL protein was significantly decreased in granule cells of the dentate gyrus in AD patients, in lines with our results from cells treated with 10 μM Aβ. The above suggested that in the different stages of AD progression, the alteration in LPL expression level may be varied, increase first and then decrease. Moreover, the disagreement in regulation of LPL expression in AD may be also associated with differences in the brain regions investigated.

In *in vitro* study, βOHB inhibited the decrease of AceH3K9 and Ace-H4K12 levels in Aβ-induced SH-SY5Y cells. ADF was also found to alleviate the decrease of Ace-H3K9 and Ace-H4K12 levels in the cerebral cortex of AD model mice. HDACs are a class of enzymes that can regulate the level of histone acetylation. Tadahiro et al found that it is βOHB not AcAc inhibits HDACs during fasting for 24 h (Shimazu et al., 2013). These findings provided vital clues that inhibition of HDACs by βOHB plays a role in regulation of ADF on LPL expression. A HDAC inhibitor (diallyl disulfide) has been reported to induce LPL expression in adipocyte cells (Lee et al., 2007). However, the HDAC inhibitor sodium butyrate suppressed LPL expression in mesenchymal stem cells (Chen et al., 2007). The above inconsistent results may be due to the differences in HDAC subtype inhibited and cells used. HDACs are divided into four groups in mammals: the zinc-dependent class I, II and IV HDACs, and the NAD^+^-dependent class III HDACs. Class I HDAC2 and HDAC3 have been found to be implicated in AD (Xu et al., 2011). In this study, ADF and βOHB were found to alleviate the increase of HDAC2/3 expression in the cerebral cortex of AD model mice and Aβ-induced SH-SY5Y cells, respectively. Interestingly, we found that the expression of LPL was elevated in HDAC3-silenced but reduced in HDAC2-silenced SH-SY5Y cells. The mechanism on the opposite effects of HDAC2/3 on the regulation of LPL expression is still unclear. It is necessary to further study whether the above opposite effect of HDAC2 and HDAC3 participates in the mechanism on the opposite effects of different concentrations of Aβ on the regulation of LPL expression in the presence or absence of βOHB.

MiR-29a has been documented to repress the expression of LPL in oxLDL-stimulated dendritic cells (Chen et al., 2011). In the present study, decreased miR-29a levels were found in the cerebral cortex of AD model mice and SH-SY5Y cells induced by 2 μM Aβ, which were alleviated by ADF and βOHB, respectively. In addition, miR-29a level was increased in 10 μM Aβ induced SH-SY5Y cells, which was also alleviated by βOHB. Further research confirmed that LPL expression was decreased in cells treated with miR-29a mimic, and increased in cells treated with miR-29a inhibitor. Moreover, we found that the expression of miR-29a was elevated in HDAC2-silenced and reduced in HDAC3-silenced SH-SY5Y cells. These findings suggest that miR-29a plays a key role in the regulation of LPL, which mediates, at least partly, the regulation of HDACs on LPL expression in AD. Further study is needed to examine the roles of HDAC2/3 in the regulatory mechanisms on miR-29a.

In summary, in the present study, ADF was observed to alleviate the increase of brain-derived LPL expression in AD model mice, which was partly mediated by βOHB. Furthermore, miR-29a was found to mediate the effect of βOHB on LPL expression, which HDAC2/3 may be implicated in the effect of βOHB on miR-29a expression. This study also found HDAC2 and HDAC3 exerted the opposite effects on the regulation of LPL and miR-29a expression levels. Further study is necessary to investigate whether other βOHB-modulated HDAC subtypes are involved in the effect on LPL. As a redox pair with AcAc, βOHB may also participate in protecting against AD via anti-oxidation pathway (Xie et al., 2015). Intermittent fasting may be utilized as a potential candidate for AD therapy and prevention. However, considering the conclusions that we obtained in regard to AD models of *in vivo* and *in vitro*, it should be prudent to extrapolate it from AD models to humans.

## Author contributions

LA conceived and designed the experiments. JZ and XL performed the experiments. AX, CJ, and YC analyzed the data. YR and YZ contributed to collection of samples and preparation of regents and materials. JZ and LA drafted the manuscript. All authors approved the final version to be published.

### Conflict of interest statement

The authors declare that the research was conducted in the absence of any commercial or financial relationships that could be construed as a potential conflict of interest.
